# Silent Fungal Invasion: A Case of Aspergillus Brain Abscesses in an Immunocompetent Older Adult Patient

**DOI:** 10.7759/cureus.86084

**Published:** 2025-06-15

**Authors:** Jamie Therese Abad, Amanda Darzi, Kashmira Wani, Jasmine Omar

**Affiliations:** 1 Internal Medicine, Henry Ford Health System, Detroit, USA

**Keywords:** aspergillus, aspergillus spp, brain abscess, cerebral aspergillosis, unusual presentations of aspergillosis

## Abstract

Brain abscesses caused by fungal pathogens are uncommon in immunocompetent individuals. An 84-year-old man presented with fever and headache. Brain imaging identified a nonspecific right frontal lesion. He returned with worsening symptoms and confusion after two weeks, with magnetic resonance imaging (MRI) revealing multiple abscesses with ring enhancement. Cultures from surgical resection and drainage samples grew *Aspergillus*. Despite antifungal therapy, his neurological condition declined. This case highlights the importance of considering fungal pathogens in older patients with nonspecific brain lesions, even without focal neurological symptoms or evidence of a primary infection.

## Introduction

Brain abscesses are focal areas of necrosis within the brain parenchyma that typically arise from an infectious process within the nearby structures or through hematogenous spread from distant organs. Brain abscesses account for approximately 8% of intracranial masses in low-resource countries and 1-2% in high-resource countries [1]. Bacterial infections are the most common cause of brain abscesses, with *Staphylococcus aureus* and viridans streptococci being the predominant associated pathogens [1]. However, fungal pathogens such as *Aspergillus* species are increasingly implicated as a cause of brain abscesses. Central nervous system (CNS) aspergillosis can be a fatal infection, with the paranasal sinuses and lungs being the most common primary sites of infection. It is more commonly seen in immunocompromised individuals, including solid organ and stem cell transplant recipients, but rarely in immunocompetent adults, especially those who have no evidence of a primary infection [2]. 

We present the case of an 84-year-old immunocompetent man who developed radiographically enlarging *Aspergillus* brain abscesses with no evidence of primary pulmonary or sinonasal involvement. This case underscores the importance of maintaining a high index of suspicion for fungal pathogens in patients with nonspecific cranial lesions suggestive of brain abscesses, even in those without overt neurological symptoms or traditional risk factors for invasive fungal disease.

## Case presentation

An 84-year-old man presented to the Emergency Department (ED) with a sharp headache located in the right temporal region. He denied any previous history of headaches. He denied any systemic symptoms such as fever, chills, or weight loss. He denied any visual disturbances, weakness, numbness, tingling, dizziness, speech changes, or light or sound sensitivity. His past medical history included Alzheimer's disease, hypertension, coronary artery disease, atrial fibrillation, and heart failure with preserved ejection fraction. He did not have diabetes mellitus and was not immunosuppressed. His medications included atorvastatin, donepezil, and valsartan.

Routine laboratory workup was unremarkable (Table 1). Computed tomography (CT) of the head was unremarkable for acute findings but showed patchy periventricular white matter hypodensities, likely related to chronic microvascular ischemic changes without evidence of acute intracranial hemorrhage or large territory infarction. He was then discharged home. He re-presented to a different ED one week later with a persistent, now dull headache and sensation of pressure behind his right eye. He again denied any systemic symptoms as well as neurological changes, including vision changes. He was noted to have tenderness in his temporal region. Routine laboratory workup with complete blood count (CBC), basic metabolic panel (BMP), troponin, and erythrocyte sedimentation rate (ESR) was within normal limits. C-reactive protein (CRP) was elevated at 1.8 mg/dL (reference range 0.000-0.744 mg/dL). CT angiography of his head and neck showed chronic ischemic changes as well as narrowing and possible occlusion of the distal right vertebral artery and stenosis involving the left vertebral artery and posterior cerebral arteries. He was recommended for further diagnostic testing on an outpatient basis. His symptoms improved with analgesics, and he was discharged home. 

**Table 1:** Initial laboratory workup. WBC: White blood cell; CRP: C-reactive protein; ESR: Erythrocyte sedimentation rate

Test	Result	Reference Range
WBC count	8.3 K/uL	3.8-10.6 K/uL
Hemoglobin	13.1 g/dL	13.5-17.0 g/dL
Hematocrit	37.6%	41-53%
Platelet count	221 K/uL	150-450 K/uL
Creatinine	0.67 mg/dL	<1.13 mg/dL
CRP	0.5 mg/dL	<0.5 mg/dL
ESR	6 mm/Hr	<20 mm/Hr

He re-presented three weeks later after a fall at home. He reported standing from a seated position, losing his balance, and falling over. He sustained a laceration on his right ear that was repaired. He was hemodynamically stable and had negative orthostatic vital signs. Routine laboratory tests, including a CBC and BMP, were within normal limits. CRP and ESR were not checked. CT of the spine showed multilevel degenerative changes and no fractures. CT of the head demonstrated a new irregular hypodensity in the right frontal region, not present on prior imaging, concerning for possible trauma, infarction, infection, or neoplasm. Given that he had no focal neurological deficits, the patient was discharged with plans for outpatient follow-up with neurology and his primary care provider. 

Approximately two weeks later, the patient presented again to the ED, this time with confusion. Laboratory results revealed mild leukocytosis with predominant neutrophils (Table 2). 

**Table 2:** Subsequent laboratory workup. WBC: White blood cell; CRP: C-reactive protein; ESR: Erythrocyte sedimentation rate

Test	Result	Reference Range
WBC count	11.6 K/uL	3.8-10.6 K/uL
Neutrophil, absolute	8.30 K/uL	1.80-7.70 K/uL
Hemoglobin	12.6 g/dL	13.5-17.0 g/dL
Hematocrit	36.8%	41-53%
Platelet count	251 K/uL	150-450 K/uL
Creatinine	0.88 mg/dL	<1.13 mg/dL
Procalcitonin	<0.20 ng/mL	<0.25 ng/mL
Lactate	1.4 mmol/L	<2.1 mmol/L
CRP	0.5 mg/dL	<0.5 mg/dL
ESR	6 mm/Hr	<20 mm/Hr

**Figure 1:**
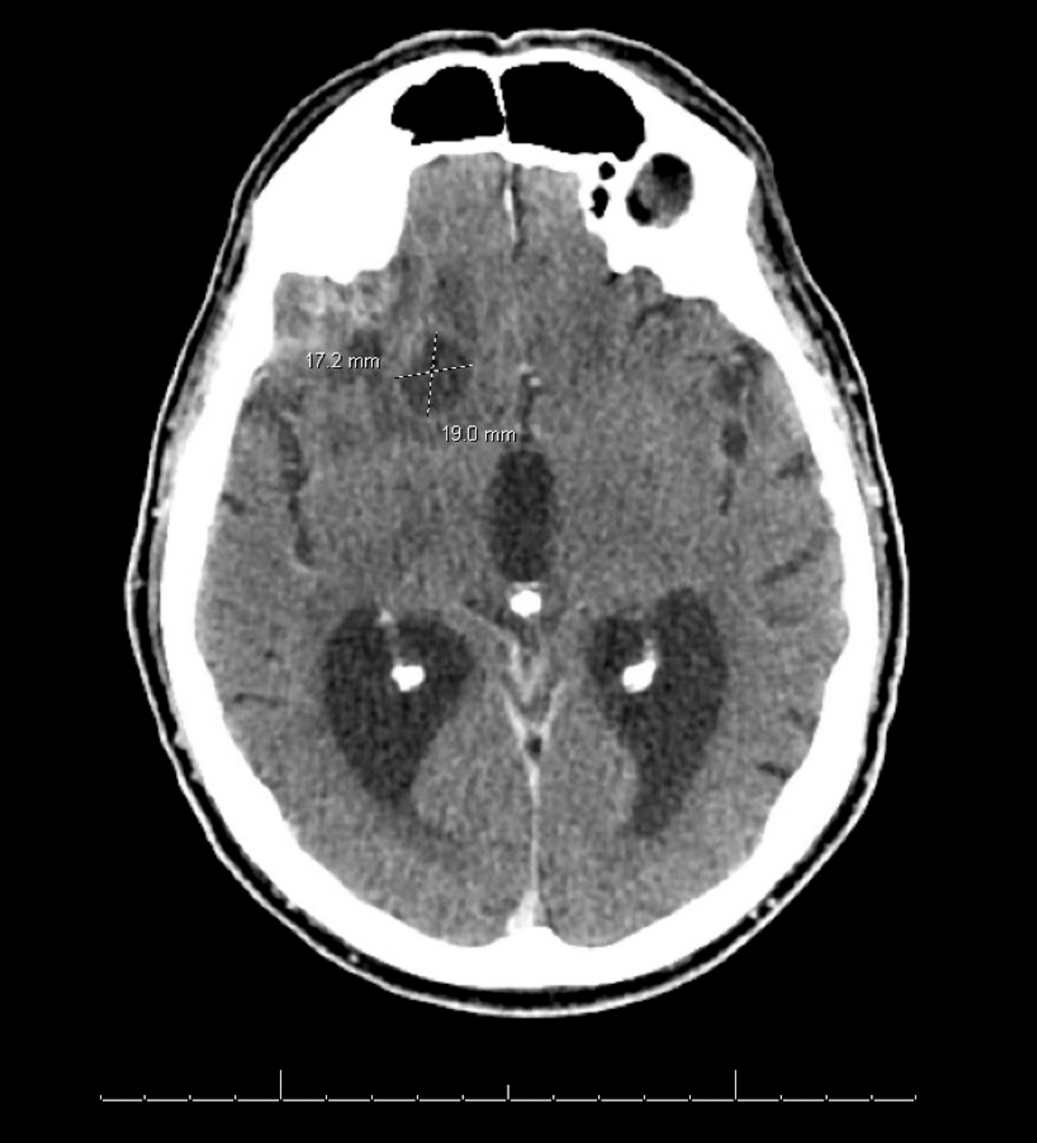
CT scan of the brain showing the right frontal lobe lesions. The scan demonstrates a low-attenuation lesion in the right frontal lobe (1.9 x 1.7 cm) with heterogeneous enhancement. No mass effect is noted. CT: Computed tomography

**Figure 2:**
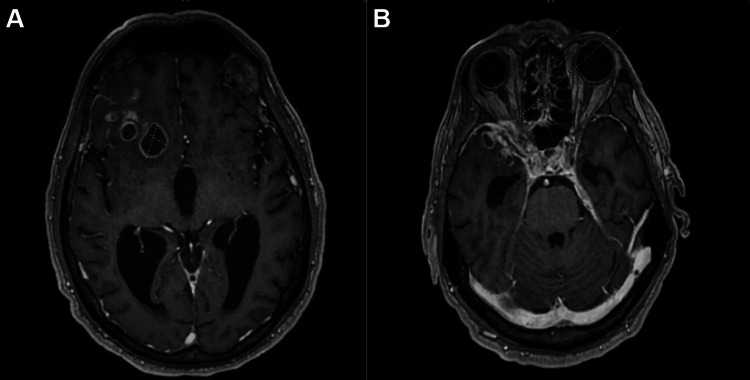
MRI of the brain confirming multiple brain abscesses. Panel (A) shows the right frontal lobe abscesses with adjacent leptomeningeal thickening (2.1 cm). Panel (B) highlights a subperiosteal abscess in the posterior right orbit with mild optic nerve mass effect. MRI: Magnetic resonance imaging

CT showed interval development of multiple low-attenuation right frontal lobe lesions extending to the periphery. The largest lesion was 1.9 x 1.7 cm with heterogenous enhancement, raising concern for infection rather than neoplasm (Figure 1). Magnetic resonance imaging (MRI) of the brain confirmed multiple right frontotemporal abscesses (up to 2.1 cm) with adjacent leptomeningeal thickening, a thin rim-enhancing subdural empyema (0.4 cm) in the right middle cranial fossa, and a 0.8 cm subperiosteal abscess in the posterior right orbit causing mild mass effect on the optic nerve (Figure 2). He was started on vancomycin, ceftriaxone, and metronidazole for broad-spectrum antimicrobial coverage and levetiracetam for seizure prophylaxis. 

The patient underwent a right frontal craniotomy with resection and evacuation of the subdural empyema and intra-axial abscesses. Drainage cultures were positive for *Aspergillus* species, and 1,3-beta-D-glucan, as determined by Fungitell assay, was elevated at 456 pg/mL (reference range positive >79 pg/mL). Therefore, liposomal amphotericin B at a dose of 5 mg/kg/day was initiated, and vancomycin was discontinued. After 10 days of treatment, liposomal amphotericin B was discontinued due to the development of acute kidney injury, and the patient was transitioned to oral voriconazole at 200 mg every 12 hours. Notably, no pulmonary or paranasal sinus source of infection was identified on additional CT imaging, and multiple sets of blood cultures remained negative. Serum *Aspergillus* galactomannan was obtained during admission and was negative (index 0.15; reference range <0.50). After nearly three weeks, the patient was discharged with plans for three to six months of voriconazole with biweekly voriconazole serum level and liver function monitoring, as well as close follow-up with infectious disease, neurosurgery, and primary care. 

However, three days after discharge, the patient presented again to the ED with persistent fever up to 39°C and new symptoms including ptosis, blurry vision, and right eye drainage. He was started on empiric vancomycin and cefepime along with voriconazole. Laboratory workup revealed worsening leukocytosis (white blood cells 21.6 K/uL) and renal impairment (creatinine 2.28 mg/dL from baseline 0.8 mg/dL). Urine cultures grew *Pseudomonas aeruginosa*, although the patient did not endorse any urinary symptoms. In light of concern for worsening abscesses without surgical source control, the infectious disease team recommended continuing voriconazole, vancomycin, and cefepime, with the addition of metronidazole for reported new sinus pain. MRI of the brain showed post-craniotomy changes, multiple intra-axial brain abscesses, and possible extension of infection into the cavernous sinus, raising concern for an unresolved empyema. Endophthalmitis and intraocular extension were ruled out after ophthalmological evaluation showed no optic nerve compromise. Around this time, the patient developed altered mentation, which continued to deteriorate despite ongoing antimicrobial therapy.

Given the extent of the infection and the patient’s high risk for complications, neurosurgeons determined that adequate source control would be difficult and that further surgical intervention would not be beneficial. The patient’s mentation continued to decline, and he was transitioned to hospice care for comfort measures. 

## Discussion

CNS aspergillosis is frequently fatal, with mortality rates remaining high despite advances in diagnosis and treatment [3]. The paranasal sinuses and lungs are typically identified as the most common primary foci of infection. In a large case series and literature review, immunosuppression was nearly universal among patients with CNS aspergillosis, with neutropenia, hematologic malignancy, solid organ transplantation, and chronic corticosteroid use as the most frequent risk factors [4]. In immunocompetent patients, CNS aspergillosis is rarer and is often a result of direct extension of infection from adjacent structures like the paranasal sinuses rather than hematogenous spread, which predominates in immunocompromised hosts. Local factors, such as prior brain injury, neurosurgical procedures, or disruption of the blood-brain barrier, can also create a permissive environment for *Aspergillus* invasion, indicating that localized immune dysfunction can substitute for systemic immunosuppression in predisposing to infection [4,5]. Notably, *Aspergillus* species are known for their angioinvasive properties, leading to cerebral infarction, hemorrhage, and subsequent abscess formation [6]. 

Interestingly, a review has shown that up to 22% of CNS aspergillosis cases had no identifiable primary infection source, underscoring the potential for fungal brain abscesses to occur in immunocompetent adults [4,7]. Our case exemplifies this phenomenon in an older immunocompetent patient without evidence of pulmonary or sinus involvement. 

Patients with *Aspergillus* brain abscesses often present with nonspecific symptoms such as headache, fever, and neurological deficits, making early diagnosis challenging. In our patient, chest radiographs showed no signs of aspergilloma in the lungs, and he had no reported respiratory symptoms that would have prompted CT chest imaging early in the presentation. MRI of *Aspergillus* brain abscesses typically reveals parenchymal lesions with ring enhancement, as seen with our patient [3]. Given the absence of a primary source, other sources of our patient’s cranial infection may have been transient fungemia from an unidentified site or a cryptic, subclinical disseminated fungal infection. Diagnostic approaches, such as cerebrospinal fluid (CSF) galactomannan antigen testing and polymerase chain reaction (PCR)-based fungal assays, although not routinely performed, may aid early diagnosis in select cases. Clinical studies have shown that CSF galactomannan testing has high sensitivity and specificity for diagnosing CNS aspergillosis, with some reports suggesting both exceed 85% at appropriate optical density index cutoffs [8]. CSF 1,3-β-D-glucan can also demonstrate good sensitivity, though it has lower specificity and is most helpful in patients with a high pre-test probability of fungal CNS infection [9]. In addition, PCR-based fungal assays may detect fungal DNA in the CSF, even in cases where cultures are negative, although their diagnostic accuracy varies and they should be interpreted within the broader clinical and laboratory context [9]. 

The lack of a clear infectious source in our patient underscores the need for a broad differential diagnosis that includes the possibility of fungal etiology in patients with frontal cranial lesions and worsening neurological symptoms, even in patients without traditional risk factors. 

Early recognition and combined antifungal and surgical treatment are key factors associated with improved outcomes in this otherwise often fatal infection. Treatment for cerebral aspergillosis often requires both surgical and medical approaches. Medical therapies alone may suffice for patients with small abscesses (<2 cm) or those accompanied by meningitis, but larger or more invasive infections typically require surgical intervention alongside antifungal therapy [5,10]. Lipid formulations of amphotericin B are reserved for cases refractory to voriconazole [9]. The rationale for this recommendation is based on clinical studies and comparative trials showing improved outcomes with voriconazole compared to amphotericin B, as well as its superior CNS penetration. Therapeutic drug monitoring of voriconazole is often recommended to ensure effective serum levels and reduce toxicity, given its variable pharmacokinetics [11,12]. Surgical resection of abscesses or contiguous infection sites is recommended when feasible, and reducing immunosuppression improves outcomes. Use of corticosteroids and intrathecal or intralesional antifungal therapy is discouraged due to limited efficacy and potential harm [1,9]. 

A case series reported five immunocompetent female patients (mean age 23 years) with isolated cerebral aspergillosis, all treated with surgery and antifungals, with a relatively favorable prognosis (80% survival) [1]. In contrast, our older patient experienced a more fulminant course despite aggressive combination therapy, suggesting that age and other host factors may worsen prognosis. 

This case emphasizes the importance of rapid multidisciplinary care for patients with complex CNS infections. Notably, early neuroimaging of brain abscesses with MRI is crucial, since CT does not have the resolution needed to detect subtle foci. The case also highlights the importance of considering fungal infection in older patients who display fever and headache that develop into neurological changes, even in the absence of typical risk factors. While *Aspergillus* brain abscesses are usually seen in immunocompromised individuals, our patient’s situation challenges our understanding of fungal pathogenesis within the CNS. But regardless of the infectious process, once an *Aspergillus* brain abscess has been identified, complete surgical infection source control is critical, and aggressive treatment and continuous monitoring are essential. 

## Conclusions

This case illustrates the potential of CNS aspergillosis, even in an immunocompetent older adult without a clear infectious source. Early recognition, prompt neuroimaging, and multidisciplinary coordination are crucial. Clinicians should maintain a high index of suspicion for fungal etiologies in patients with progressive neurological symptoms and atypical imaging findings, as delays in diagnosis and treatment can result in irreversible neurologic decline and poor outcomes. 

## References

[1] Hall WA, Mesfin FB (2024). Brain abscess. StatPearls [Internet].

[2] Bokhari R, Baeesa S, Al-Maghrabi J, Madani T (2014). Isolated cerebral aspergillosis in immunocompetent patients. World Neurosurg.

[3] Shamim MS, Enam SA, Ali R, Anwar S (2010). Craniocerebral aspergillosis: a review of advances in diagnosis and management. J Pak Med Assoc.

[4] Kourkoumpetis TK, Desalermos A, Muhammed M, Mylonakis E (2012). Central nervous system aspergillosis: a series of 14 cases from a general hospital and review of 123 cases from the literature. Medicine (Baltimore).

[5] Husain S, Camargo JF (2019). Invasive aspergillosis in solid-organ transplant recipients: guidelines from the American Society of Transplantation Infectious Diseases Community of Practice. Clin Transplant.

[6] Patterson TF, Thompson GR 3rd, Denning DW (2016). Practice guidelines for the diagnosis and management of aspergillosis: 2016 update by the Infectious Diseases Society of America. Clin Infect Dis.

[7] Ma Y, Li W, Ao R, Lan X, Li Y, Zhang J, Yu S (2020). Central nervous system aspergillosis in immunocompetent patients: case series and literature review. Medicine (Baltimore).

[8] Chong GM, Maertens JA, Lagrou K, Driessen GJ, Cornelissen JJ, Rijnders BJ (2016). Diagnostic performance of galactomannan antigen testing in cerebrospinal fluid. J Clin Microbiol.

[9] Lehrnbecher T, Rath PM, Attarbaschi A (2019). Galactomannan and PCR in the central nervous system to detect invasive mold disease - a retrospective analysis in immunocompromised children. Sci Rep.

[10] Miceli MH (2019). Central nervous system infections due to Aspergillus and other hyaline molds. J Fungi (Basel).

[11] Luong ML, Al-Dabbagh M, Groll AH, Racil Z, Nannya Y, Mitsani D, Husain S (2016). Utility of voriconazole therapeutic drug monitoring: a meta-analysis. J Antimicrob Chemother.

[12] Pappas PG, Kauffman CA, Andes DR (2016). Clinical practice guideline for the management of candidiasis: 2016 update by the Infectious Diseases Society of America. Clin Infect Dis.

